# Scientific production of an oral implantology journal: a 5-year bibliometric study

**DOI:** 10.1007/s11192-023-04696-4

**Published:** 2023-04-24

**Authors:** Julián Espinosa-Giménez, Vanessa Paredes-Gallardo, María Dolores Gómez-Adrián, Carlos Bellot-Arcís, Verónica García-Sanz

**Affiliations:** 1 Stomatology Department, University of Valencia, Valencia, Spain; 2 Department of Surgery and Oral Implantology, Universidad Católica de Valencia, Valencia, Spain

**Keywords:** Bibliometric analysis, Scientific production, Citation count, Implantology

## Abstract

Oral implantology is a science in constant evolution, with a considerable number of articles being published every year in scientific journals. Publications can be analyzed through bibliometric analysis, thus observing the evolution and trends of the articles published in the journal. To evaluate, through bibliometric analysis, the scientific production of Clinical Implant Dentistry and Related Research (CIDRR) and its evolution and trends in the last 5 years (2016–2020).All articles published in CIDRR in the last 5 years were reviewed and classified according to the year of publication, volume, number, the number of authors, demographic data of the first and last author, the geographical scope of the article, the number of affiliations of the authors, research topic, type of study, and study design. The association between these variables and citation counts was also analyzed. 599 articles were analyzed. 77.4% were authored by 4–6 authors, obtaining 78.4% from 1 to 3 different affiliations. Male researchers predominated in both the first and last authorship. China showed the highest number of publications when comparing the origin of the authors’ affiliations individually; however, most researchers (40.9%) were from the European Union (EU)-Western Europe area. The most studied topic was the implant/abutment design/treatment of the surface (19.1%). Clinical research articles accounted for 92.99% of the publications, of which cross-sectional observational studies prevailed (21.7%). The presence of articles from the United States of America-Canada and EU-Western Europe was positively correlated with the impact factor. This study revealed an increasing trend in Asian research production, particularly Chinese, whereas production of European origin showed a decrease. Clinical studies increased their relative weight to the detriment of translational ones. A growing tendency in the relative weights of female authors was appreciated. Journal citations were associated with certain study variables.

## Introduction

Since the emergence of modern implant dentistry (Brånemark et al, 1977) and its acceptance by the dental community after the 1982 Toronto Conference on Osseointegration (Zarb, 1983), dental implantology has been a demanded therapy by patients to replace missing teeth that have demonstrated high levels of clinical success (Heydecke et al., 2003).

As consequence, an increasing world population is being treated with dental implants (Elani et al., 2018; Schimmel et al., 2017) making them an interesting matter for scientific research.

With the expansion of dental implant literature in specialized journals through the years, scientific articles were collected in databases such as the Web of Science (WoS) for its accessibility to the scientific community. From the citations collected in WoS for the articles indexed in this database, the Journal Citation Reports (JCR), a research tool recognized by the scientific community, elaborates metrics to assess the impact of the journals, being the best known the Journal Impact Factor (JIF) (Garfield, 2007).

According to JCR, 91 journals are included in the "Dentistry, Oral Surgery & Medicine" category in the 2020 data release due to their JIF, of which 9 directly focus their research on oral implantology.

The present study focused on a single journal, Clinical Implant Dentistry and Related Research (CIDRR) as a representative journal related to implant dentistry. Following the JCR 2020 classification, CIDRR was listed in the 18th position out of 91 journals.

Dental implantology, as a part of Dentistry, follows the principles of Evidence-Based Medicine (Kashi et al., 2013), which classifies its research methods following an established pyramid of hierarchy to reflect the reliability of application to clinical practice. Systematic reviews and meta-analyses can be found at the pinnacle of them, followed in descending order by randomized controlled trials (RCTs), cohort, case-controlled and cross-sectional studies, case reports, and expert opinions. (Wilson et al., 2021). Animal research would be placed at the base of them (Lee, 2014).

Analyses of published literature can be achieved through bibliometrics, a tool that has proved effective in evaluating scientific activity considering statistical methods (Haustein & Larivière, 2015).

Several bibliometric studies in the field of dentistry have been published in recent decades (Ahmad et al., 2020; Yeung & Ho, 2019), some of which are related to oral implantology (Fardiet al., 2017; Lorusso et al., 2020; Tarazona et al., 2017b), studying certain indicators, such as funding (Alonso-Arroyo et al., 2019), level of evidence (Wu et al., 2020), or geographic scope (Tarazona et al., 2017a). However, bibliometric studies focused on the evolution and trends of the articles published in a single determined journal are scarce (Ahmadet al., 2019; Estrela et al., 2020; Alhajj et al., 2021) and none of the reviewed literature refers to implant-related journals.

For these reasons, the present study aimed to perform a complete and extensive bibliometric analysis of all the articles published in CIDRR over 5 years (2016–2020) to evaluate the tendencies, topics, and evolution of the lines of research in oral implantology and to analyze the possible correlations between citation count and the evaluated parameters. With these findings, the study aims to obtain a broad picture of the current state of the studies in dental implantology, prominent authors and institutions, and the most prevalent world regions studying this subject.

## Methods

The CIDRR website was accessed to obtain all issues published between 2016 and 2020, and all articles included in them were analyzed. In the present study, letters to the editor, replies, and Corrigendum were excluded as they were determined only to include conventional articles of high scientific value, to avoid duplications in author and article-related parameters.

Three types of parameters: issue-related, author-related, and article-related, were registered.

The issue-related parameters included the following bibliometric indicators: year of publication of the journal, volume and issue number, the pages of the article in the volume, and the title of the article.

For the author-related parameters, the Scopus database was used to recover the author's data. This study included: the number of authors signing the article; the first and last author's name, institution (in cases where an author collaborated with more than one institution, only one was considered. This institution was selected considering the institutions of the rest of the authors of the manuscript), and sex; the first and last author's affiliation (surgery, periodontics, prosthodontics, other, none, or mixed); the first and last author's country of origin, assigned to a geographic world region (USA-Canada, European Union-Western Europe, Rest of America, Rest of Europe, Eastern Asia, Africa, Rest of Asia, Oceania-Pacific Islands); the geographic collaboration index (Local when all authors were registered in the same institution, National when all the authors were registered in institutions in the same country, International when authors were registered in institutions of different countries); and the H-index of top first and last authors, as searched in Scopus on August 31, 2021.

Finally, the article-related parameters included: the topic of the article, where papers could be registered at more than one topic owing to their theme (Table 1); type of study (Clinical research, Systematic Reviews and Meta-analysis, Narrative review, Case Report, Other), and the Study design only in research articles based on Farjo et al.'s classification (Farjo et al., 2015).

**Table 1:** Main topics of the articles

Main Topics	
T1	Implant/abutment design/treatment of surface
T2	Bone regeneration/expansion
T3	Prosthesis
T4	Sinus elevation
T5	Special care patients, Elderly patients, Patients under special conditions (tobacco, bruxism, other medications)
T6	Immediate implantology
T7	Periimplantitis/Biofilm
T8	Implant Review/followment/Maintenance
T9	Conventional Implant surgery/Implant integration
T10	Immediate/Early implant loading
T11	Guided surgery
T12	Image diagnosis (CBCT, OPT, US, etc.)
T13	Implant primary stability
T14	Implant marginal bone loss
T15	Implant Failure/Fracture
T16	Anatomy
T17	Treatment satisfaction
T18	Gingiva, papillae, pink aesthetics
T19	All-on-four technique
T20	Soft tissue regeneration
T21	Zygomatic implants
T22	Complications
T23	General state of implantology/Bibliometrics
T24	Bone volumetric changes
T25	Analysis of performed treatments
T26	Piezoelectric surgery
T27	Socket shield technique
T28	Patient’s perception/information
T29	Use of LASER
T30	Use of Biphosphonates/Monoclonal antibody
T31	Digital work-flow
T32	Inferior Alveolar Nerve Lateralization
T33	Pain

For every studied year, the number of citations and impact factor were collected from the JCR website (Web of Science, Clarivate) to obtain correlations with the rest of the parameters studied. Web of Science website was accessed on August 31, 2021, to retrieve all the information. Both impact factor and citations were correlated with journal data for the same year.

### Statistical analysis

A broad statistical analysis was applied to these data and possible correlations between the measured parameters and the number of citations received by the CIDRR each year.

The categorical variables (such as topic of the article or type of study) were described through absolute and relative frequencies (in percentages). For the quantitative variables (number of authors and affiliations), means, standard deviations, medians and ranges were presented. The descriptive analysis was carried out for the total sample of articles differentiated by the year of publication.

Given that the selection of articles was exhaustive, the work sample corresponded exactly to the total population of articles between 2016 and 2020 in CIDRR; therefore, the inferential analysis was meaningless.

The correlations of the number of citations and the impact factor of the journal with the different study variables were analyzed using Spearman's correlation coefficient.

## Results

The sum of all research, systematic reviews/meta-analyses, narrative reviews, and case reports were 599 articles. During this 5-year analysis review, no other types of studies other than the previously mentioned were published.

### Issue-related parameters

The distribution of articles was homogeneous from 2016 to 2018, at approximately 20% annually. However, the number of publications in 2019 (158 articles, 26.2%) was higher than in 2020 (82 articles, 13.6%).

### Author-related parameters

The average number of authors per article was 4.9 ± 1.5. A total of 77.4% of the articles were authored by four to six authors. Ten studies were authored by a single author (1.67%).

Regarding the first author, 488 authors from 242 institutions were identified. Male first authors were more prominent than females (72.3% to 27.7%).

In the case of the last authorship, results differed slightly: 403 authors from 227 institutions. Compared to first authorships, the predominance of male last authors was even more notable over female authorship (79.9% to 20.1%).

Table 2 lists the authors with a larger number of publications and their H-indices (searched in Scopus on August 31, 2021) and the top five institutions with a larger number of publications.

**Table 2:** Top authors with their H-index and top five institutions

Top first authorship				
Author	Institution/s	Country	Number of papers/total percentage	H-index
Jemt, Torsten	University of Gothenburg/Brånemark Clinic	Sweden	8 (1.34%)	62
Chrcanovic, Bruno Ramos	University of Malmö	Sweden	5 (0.83%)	35
Al-Aali, Khulud Abdulrahman	Princess Nourah Bint Abdulrahman University	Saudi Arabia	5 (0.8%)	10
Albrektsson, Tomas	University of Gothenburg	Sweden	4 (0.67%)	89
Thoma, Daniel S	University of Zurich	Switzerland	4 (0.67%)	35
Maló, Paulo	Maló Clinic	Portugal	4 (0.67%)	26
Yu, Huajie	Peking University	China	4 (0.67%)	6

Top last authorship				
Author	Institution/s	Country	Number of papers/ Total percentage	H-index
Abduljabbar, Tariq	King Saud University	Saudi Arabia	11 (1.84%)	20
Wennerberg, Ann	University of Gothenburg	Sweden	10 (1.67%)	65
Javed, Fawad	Stony Brook University/ University of Rochester	USA	9 (1.5%)	38
De Bruyn, Hugo	Ghent University	Belgium	8 (1.34%)	44
Del Fabbro, Massimo	University of Milan/IRCCS Orthopedic Institute Galeazzi	Italy	6 (1%)	46
Wu, Yiqun	Shanghai Jiao Tong University	China	6 (1%)	15
Lin, Ye	Peking University	China	6 (1%)	13
Qiu, Lixin	Peking University	China	6 (1%)	8
Akram, Zohaib	Ziauddin University	Pakistan	5 (0.83%)	21
Vandeweghe, Stefan	Ghent University	Belgium	5 (0.83%)	20

Top five institutions by first authorship				
Institution		Country		Number of papers/ Total percentage
Private Practice		**–**		26 (4.34%)
Ghent University		Belgium		21 (3.5%)
University of Gothenburg		Sweden		19 (3.17%)
Peking University		China		18 (3%)
King Saud University		Saudi Arabia		18 (3%)

Top five institutions by last authorship				
Institution		Country		Number of papers/ Total percentage
Ghent University		Belgium		20 (3,33%)
University of Gothenburg		Sweden		19 (3,17%)
Peking University		China		17 (2,84%)
Shanghai Jiao Tong University		China		16 (2,67%)
University of Groningen/King Saud University		Netherlands/Saudi Arabia		15 (2,5%)

The H-index of the most prolific authors ranged from 6 to 89.

**Fig. 1:**
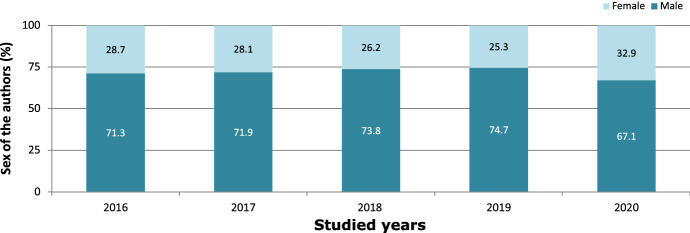
First author sex evolution

The 5-year evolution indicated a growing tendency in the relative weight of the female researchers' population, as shown in Fig. 1.

The average number of affiliations per article was 2.5 ± 1.3. Approximately 78.4% of articles included between one and three different affiliations. Approximately 32% of the first and last authors were affiliated with the surgery category, whereas another one-third was divided between prosthodontics and periodontics. The remaining one-third was divided between combinations of the previous three, other types, or none at all.

**Fig. 2:**
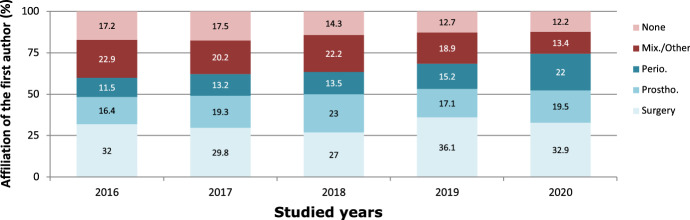
First author affiliation evolution

An increase in the relative weight of periodontology affiliation was appreciated, whereas surgery and prosthodontics showed irregular percentages with no clear tendency (Fig. 2).

**Fig. 3:**
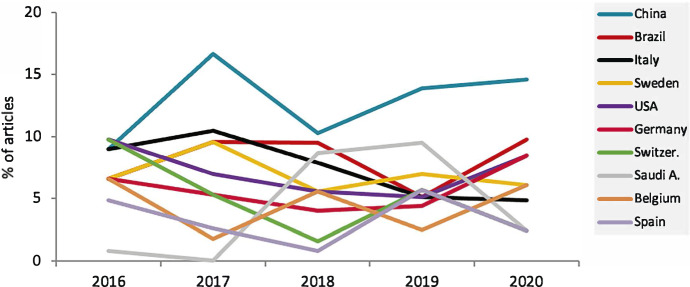
Country (first author) evolution 2016–2020

Regarding the country of origin of the researcher's institutions, China produced the most articles in both the first and last authorships (12.8% and 12.5%, respectively), followed by Brazil (7.8%), Italy (7.5%), Sweden (7%), and the United States of America (USA, 7%). In the last authorship, however, US institutions occupied the second position in the list (9.1%) (Fig. 3).

In 2016, China was at the same level in the number of publications as other countries; however, in 2020, with a higher percentage, it distinguished itself as the predominant country. Among the irregular patterns of most countries, Switzerland and Italy stood out with a clearer decreasing trend. On the other hand, Saudi Arabia, which started the series with a testimonial production of publications, had a considerable increase during the years 2018–2019, with nearly 10% of the produced articles.

As for the geographic world regions, the majority of the research was performed in the EU-Western Europe area among both the first and last authorship (40.9% and 42.1%, respectively), followed by Eastern Asia (21.6% and 21.3%, respectively). The region with the smallest number of publications was Oceania-Pacific Islands (1.7% and 1.7%), as the articles came only from Australia and New Zealand, followed by Africa (3% and 2.9%, respectively), with articles exclusively from Egypt and South Africa.

Despite not being a geographical world region itself, several publications were produced in the emergent BRICS countries (Brazil, Russia, India, China, and South Africa), with only the combination of China and Brazil meaning 24,4% of the published articles in 2020.

**Fig. 4:**
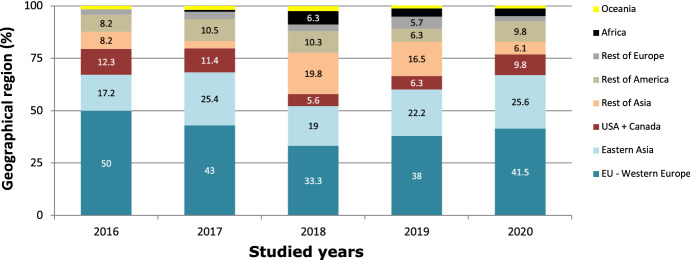
Geographic World Region evolution 2016–2020

According to the first author's geographic affiliation, the weight of the countries in the EU-Western Europe decreased during this period, whereas Asian production (Eastern Asia + rest of Asia) increased. The 2018 distribution was noteworthy, with a highly important weight from the rest of Asia (19.8%), which implied that the three leading areas barely exceeded 55% of the total articles (Fig. 4).

Considering the geographic collaboration index, a certain predominance of the local scope was observed, resulting in 39.5% of the articles with all authors coming from the same institution. Tendency showed that local-type publications had been increasing their relevance, whereas collaborations between institutions settled in different countries decreased.

### Article-related parameters

Regarding the main topic of the articles, the "implant/abutment design/treatment of surface" was the most recurrent one (19.1%), followed by "bone regeneration/expansion" procedures (15.1%) and "prosthetic" (10%). Table 3 shows the 10 more prevalent topics, which exceeded 5% of the publications.

**Table 3:** Most prevalent topics, type of study and clinical research study design distribution

	2016	2017	2018	2019	2020	Total
Most prevalent topics						
Implant/abutment design/treatment of surface	23 (18.9%)	21 (18.4%)	25 (19.8%)	30 (19.36%)	16 (19.5%)	115 (19.1%)
Bone regeneration/expansion	16 (13.1%)	19 (16.7%)	16 (12.7%)	26 (16.77%)	14 (17.1%)	91 (15.1%)
Prosthesis	13 (10.7%)	5 (4.4%)	10 (7.9%)	20 (12.9%)	12 (14.6%)	60 (10%)
Sinus elevation	13 (10.7%)	12 (10.5%)	9 (7.1%)	13 (8.39%)	2 (2.4%)	49 (8.1%)
Special care, elderly and patients under special conditions	12 (9.8%)	6 (5.3%)	11 (8.7%)	13 (8.39%)	5 (6.1%)	47 (7.8%)
Immediate implantology	8 (6.6%)	9 (7.9%)	5 (4%)	9 (5.81%)	10 (12.2%)	41 (6.8%)
Periimplantitis/Biofilm	6 (4.9%)	3 (2.6%)	8 (6.3%)	16 (10.32%)	7 (8.5%)	40 (6.6%)
Implant review/followment/maintenance	8 (6.6%)	8 (7%)	9 (7.1%)	5 (3.22%)	5 (6.1%)	35 (5.8%)
Conventional Implant surgery/ integration	11 (9%)	5 (4.4%)	9 (7.1%)	5 (3.22%)	3 (3.7%)	33 (5.5%)
Immediate/Early implant loading	5 (4.1%)	7 (6.1%)	5 (4%)	7 (4.52%)	7 (8.5%)	31 (5.1%)
Type of study						
Clinical Research	111 (90.98%)	110 (96.49%)	114 (90.48%)	144 (92.9%)	78 (95.12%)	557 (92.99%)
Systematic Review & Metaanalyses	9 (7.38%)	3 (2.63%)	12 (9.52%)	8 (5.16%)	3 (3.66%)	35 (5.84%)
Narrative review	2 (1.64%)	0 (0%)	0 (0%)	3 (1.94%)	1 (1.22%)	6 (1%)
Clinical Case	0 (0%)	1 (0.88%)	0 (0%)	0 (0%)	0 (0%)	1 (0,17%)
Clinical research study design						
Basic	2 (1.8%)	0 (0%)	5 (4.4%)	5 (3.5%)	1 (1.3%)	13 (2.3%)
+Materials	1 (0,9%)	0 (0%)	2 (1.8%)	1 (0.7%)	0 (0%)	4 (0.7%)
+ Cell	1 (0.9%)	0 (0%)	3 (2.6%)	4 (2.8%)	1 (1.3%)	9 (1.6%)
− Translational	34 (30.6%)	10 (9.1%)	23 (20.2%)	26 (18.1%)	15 (19.2%)	108 (19.4%)
+ Human	4 (3.6%)	0 (0%)	2 (1.8%)	1 (0.7%)	3 (3.8%)	10 (1.8%)
+ Animal	23 (20.7%)	6 (5.5%)	9 (7.9%)	13 (9%)	6 (7.7%)	57 (10.2%)
+ Theoretical	7 (6.3%)	4 (3.6%)	12 (10.5%)	12 (8.3%)	6 (7.7%)	41 (7.4%)
− Clinical	75 (67.5%)	100 (90.9%)	86 (75.4%)	113 (78.5%)	62 (79.5%)	436 (78.3%)
+ Cotrolled trials	26 (23.4%)	51 (46.4%)	30 (26.3%)	44 (30.6%)	24 (30.8%)	175 (31.4%)
*Randomized	17 (15.3%)	28 (25.5%)	20 (17.5%)	25 (17.4%)	18 (23.1%)	108 (19.4%)
*Non-Randomized	9 (8.1%)	23 (20.9%)	10 (8.8%)	19 (13.2%)	6 (7.7%)	67 (12%)
+ Observational	49 (44.1%)	49 (44.5%)	56 (49.1%)	69 (47.9%)	38 (48.7%)	261 (46.9%)
*Cohort	21 (18.9%)	21 (19.1%)	14 (12.3%)	20 (13.9%)	13 (16.7%)	89 (16%)
*Case control	2 (1.8%)	8 (7.3%)	7 (6.1%)	8 (5.6%)	1 (1.3%)	26 (4.7%)
*Case series	6 (5.4%)	3 (2.7%)	5 (4.4%)	7 (4.9%)	4 (5.1%)	25 (4.5%)
*Cross-sectional	20 (18%)	17 (15.5%)	30 (26.3%)	34 (23.6%)	20 (25.6%)	121 (21.7%)

The majority (92.99%) were clinical research studies, followed by systematic reviews and meta-analyses (5.84%) and narrative reviews (1%). Case reports were testimonial, with only one study in 5 years (0.17%), as shown in Table 3.

**Fig. 5:**
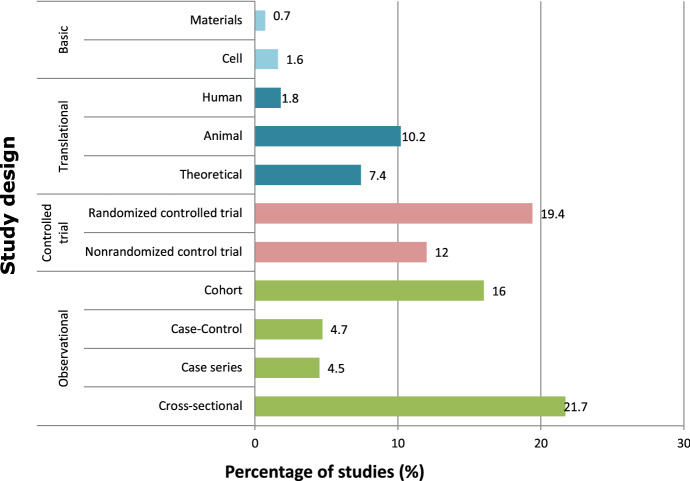
Percentage of studies according to their design

Considering the design of clinical research studies only, and according to the Farjo classification, which can be consulted in Appendix 1, basic studies accounted for 2.3% of the total, translational studies for 19.4%, and the vast majority (78.3%) were clinical, divided into controlled trials (31.4%) and observational studies (46.9%). Randomized studies were the most prevalent type of clinical controlled trial (19.4%), and cross-sectional studies were the most predominant in observational studies (21.7% of all research articles) (Fig. 5).

In general, clinical studies increased their relative weight over the years. They did this to the detriment of translational studies, as shown in Table 3. Translational studies in animals decreased the most (from 20.7% in 2016 to 7.7% in 2020), whereas cross-sectional observational studies increased substantially (from 18% in 2016 to 25.6% in 2020).

Regarding the association between the number of cites/impact factors and the study parameters, some correlations were found (Table 4). Positive correlations were found between impact factors/citations and articles from USA-Canada and EU-Western Europe, and for topics, such as sinus elevation, analysis of performed treatments, and complications among others. By contrast, negative correlations were observed for articles from Eastern Asia, Africa, or the rest of Europe or topics, such as peri-implantitis/biofilm, prosthesis, or implant primary stability, among others. Clinical observational studies were negatively correlated to citation count.

**Table 4:** Associations between number of cites/impact factor and study variables

	Citation count	Impact factor
Geographic world zone		
USA—Canada	*r* = 0.60; *p* = 0.285	***r* = 0.80;** ***p* = 0.104**
EU—Western Europe	*r* = 0.60; *p* = 0.285	***r* = 0.80;** ***p* = 0.104**
Rest of America	*r* = 0.20; *p* = 0.747	*r* = 0.10; *p* = 0.873
Rest of Europe	*r* = 0.10; *p* = 0.873	*r* = − *0.80;* *p* = *0.104*
Eastern Asia	*r* = − *0.70;* *p* = *0.188*	*r* = − 0.10; *p* = 0.873
Africa	*r* = − 0.60; *p* = 0.285	*r* = *− 0.80;* *p* = *0.104*
Rest of Asia	*r* = -0.10; *p* = 0.873	*r* = −0.50; *p* = 0.391
Oceania – Pacific islands	*r* = 0.60; *p* = 0.285	*r* = − 0.20; *p* = 0.747
Type of study		
Clinical Research	*r* = − 0.20; *p* = 0.747	*r* = 0.10; *p* = 0.873
Systematic review and meta-analysis	*r* = 0.20; *p* = 0.747	*r* = − 0.10; *p* = 0.873
Narrative review	*r* = − 0.15; *p* = 0.805	*r* = − 0.05; *p* = 0.935
Case report	*r* = 0.35; *p* = 0.559	*r* = 0.00; *p* = 1.000
Clinical research study design		
Basic	*r* = − 0.10; *p* = 0.873	*r* = − 0.50; *p* = 0.391
Translational	*r* = 0.30; *p* = 0.624	*r* = 0.50; *p* = 0.391
Clinical Controled Trials	*r* = − 0.40; *p* = 0.505	*r* = -0.20; *p* = 0.747
Clinical Observational	*r* = *− 0.70*; *p* = *0.188*	*r* = − 0.50; *p* = 0.391
Topic		
Periimplantitis/Biofilm	*r* = − *0.80*; *p* = *0.104*	r = − 0.50; p = 0.391
Sinus elevation	***r* = 0.90;** ***p* = 0.037**	*r* = 0.30; *p* = 0.624
Implant/abutment design/treatment of surface	*r* = − 0.50; *p* = 0.391	*r* = − 0.30; *p* = 0.624
Use of Biphosphonates/Monoclonal antibody	*r* = 0.67; *p* = 0.215	*r* = 0.45; *p* = 0.450
Prosthesis	*r* = *-0.70;* *p* = *0.188*	*r* = 0.10; *p* = 0.873
Conventional Implant surgery/Implant integration	***r* = 0.80;** ***p* = 0.104**	*r* = 0.60; *p* = 0.285
Immediate/Early implant loading	*r* = -0.20; *p* = 0.747	*r* = 0.10; *p* = 0.873
Implant Failure/Fracture	*r* = 0.40; *p* = 0.505	*r* = 0.20; *p* = 0.747
Implant Review/followment/Maintenance	***r* = 0.80;** ***p* = 0.104**	*r* = 0.60; *p* = 0.285
Bone regeneration/expansion	*r* = − 0.40; *p* = 0.505	*r* = −0.20; *p* = 0.747
Bone volumetric changes	*r* = 0.34; *p* = 0.581	*r* = − 0.22; *p* = 0.718
Image diagnosis (CBCT, OPT, US, etc.)	*r* = 0.30; *p* = 0.624	*r* = -0.50; *p* = 0.391
Special care patients, Elderly patients, Patients under special conditions (tobacco, bruxism, other medications)	***r* = 0.90;** ***p* = 0.037**	*r* = 0.30; *p* = 0.624
Implant marginal bone loss	*r* = 0.45; *p* = 0.450	*r* = 0.22; *p* = 0.718
Pain	–	–
Guided surgery	*r* = − 0.50; *p* = 0.391	*r* = 0.20; *p* = 0.747
Analysis of performed treatments	***r* = 0.71;** ***p* = 0.182**	***r* = 0.71;** ***p* = 0.182**
Gingiva, papillae, pink aesthetics	*r* = −0.41; p = 0.493	*r* = −0.41; *p* = 0.493
Patient’s perception/information	*r* = − 0.35; *p* = 0.559	*r* = *−0.71;* *p* = *0.182*
Immediate implantology	*r* = 0.10; *p* = 0.873	*r* = 0.50; p = 0.391
Soft tissue regeneration	*r* = *− 0.89;* *p* = *0.041*	*r* = − 0.11; *p* = 0.858
General state of implantology/Bibliometrics	*r* = −0.60; p = 0.285	*r* = − 0.30; *p* = 0.624
Zygomatic implants	*r* = -0.11; *p* = 0.858	*r* = 0.22; *p* = 0.718
Inferior Alveolar Nerve Lateralization	–	–
Treatment satisfaction	*r* = 0.20; *p* = 0.741	*r* = − 0.56; *p* = 0.322
Implant primary stability	*r* = 0.10; *p* = 0.873	*r* = *−0.80;* *p* = *0.104*
Complications	*r* = **0.71;** ***p* = 0.182**	*r* = **0.71;** ***p* = 0.182**
Use of LASER	*r* = − 0.45; *p* = 0.450	*r* = 0.34; *p* = 0.581
Socket shield technique	*r* = 0.00; *p* = 1.000	*r* = − 0.35; *p* = 0.559
Piezoelectric surgery	*r* = 0.00; *p* = 1.000	*r* = − 0.35; *p* = 0.559
Anatomy	*r* = **0.70;** ***p* = 0.188**	*r* = − 0.10; *p* = 0.873
Digital work-flow	–	–
All-on-four technique	*r* = 0.21; *p* = 0.741	*r* = − 0.56; *p* = 0.322

Bold means positive correlation (*r* ≥ 0.7) and italic means negative correlation (*r* ≤ −0.7)

*r* = Spearman correlation coefficient, *p* = *p*-value

### Discussion

Since the acceptance of dental implants by the scientific community at the 1982 Toronto Conference of Osseointegration (Zarb, 1983), scientific production in specialized journals has been increasing annually, placing several of these journals in high positions in the JCR list. Among them is CIDRR, a journal that has already been studied in different bibliometric studies (Jayaratne et al., 2015; Pommer et al., 2016).

In the present bibliometric study, an exhaustive analysis of all the articles published by the journal for 5 years (2016–2020) was conducted. The distribution of articles was homogeneous in the years 2016–2018 (20%), increased in 2019 (26.2%), and decreased in 2020 (13.6%). The reason for the larger number of publications in 2019 can be explained by the fact that the CIDRR published a special issue in March. The 2020 reduction in production might be related to the fact that the journal activity was reduced during the coronavirus disease (COVID-19) pandemic breakdown.

Several bibliometric studies have been performed in the dental scientific literature. A database search may show results in different areas, such as endodontics (Adnan & Ullah, 2018), periodontics (Ahmad et al., 2020), orthodontics (Tarazona-Alvarez et al., 2019), oral medicine (Liu et al., 2020), or dental implantology (Fardi et al., 2017), among others.

However, bibliometric studies tend to focus on certain indicators, such as funding (Alonso-Arroyo et al., 2019), level of evidence (Wu et al., 2020), or geographic scope (Tarazona et al., 2017a).

Bibliometric studies that focus on a single journal analysis are scarcer (Ahmad et al., 2019; Estrela et al., 2020; Alhajj et al., 2021), and very few studies have performed an exhaustive analysis of all published articles to assess trends (Aura-Tormos et al., 2019), as performed in the present study.

During the analysis of the data obtained in the 5-year evolution of CIDRR, certain trends were observed. A strong prevalence of male researchers was estimated, both in the first (72.3%) and last (79.9%) authorship. However, a growing tendency in the relative weight of female authors was shown, particularly in the first authors from 2019 to 2020. (Fig. 1). This trend has also been reported in other studies (Aura-Tormos et al, 2019; Li et al., 2019).

It has been observed that, in general terms, first authorship researchers are generally young professionals, who in many cases present lines of research related to their final degree project, master's, or doctoral thesis; the last authorship researcher generally refers to the department head or director of the work, generally a senior researcher (Tarkang et al., 2017). The greater presence of female researchers in the first authorship could be a consequence of the fact that the number of female professionals has grown in the dental profession in recent decades (Whelton & Wardman, 2015), whereas its lower presence as last authorship researchers could be a consequence of earlier stages, where the prevalence of female professionals was lower.

Regarding the evolution of the author's affiliation, an increase in the relative weight of the affiliation in periodontology was observed, whereas the authors assigned to areas of surgery and prosthodontics showed more stable values (Fig. 2). This may be due to the high number of publications related to the treatment of peri-implantitis or review and maintenance of implants (6.6% and 5.8%, respectively, of the articles analyzed in our review, 12.4% in total). Nevertheless, the present study found a higher percentage of studies related to implant/abutment design or treatment of the surface (19.1%) and bone regeneration/expansion (15.1%) (Table 3).

Considering the country of affiliation of the first author's institution, it was observed that in 2016, the research production of Chinese origin was at the same level as other countries; however, in 2020, with a higher percentage, China distinguished itself as the predominant country in terms of the number of articles. (Fig. 3) The increase in Chinese research production and its increase in importance in the scientific field has been highlighted in other disciplines (Xie & Freeman, 2019). In the case of CIDRR, this was particularly evident in Issue 3 of 2019 (Becker, 2019), which was entirely dedicated to publications of Chinese origin. In contrast, European countries, such as Switzerland and Italy, showed a clearer decreasing pattern in their number of publications, while other countries as Saudi Arabia began the series with a small number of publications and increased them considerably in the last studied years. This specific case may be related to the fact Saudi Arabia has done efforts to improve its investment in research during the last years (Saquib, 2018).

The same trend was observed when analyzing the publication of articles according to their geographical world region, the EU-Western Europe block contributing with the highest number of articles during the 5 years, although with a decreasing tendency. In contrast, the Asian block increased its relative research production (Fig. 3).

These tendencies were also found when analyzing authors and institutions with the largest number of publications (Table 2). European authors were the majority in the case of the first authorship; however, a considerable number of Chinese last authors (3/10) was reported. Approximately 50% of most publishing institutions had a European origin, whereas the other 50% were Asian in origin.

The fact that BRICS, as economic emergent countries, meant a predominant region itself, with around 25% of produced articles, could be attributed to the investment in innovation and research by their governments (Altbach, 2013; Niemczyk, 2020). This may also explain the presence of South Africa as the only country representative of the African region in combination with Egypt, a country which produced 2.7% of all the studied articles. Concerns in Egyptian research have already been noted by studies (Goell, 2012).

The findings of the present research are in concordance with Evidence-Based Medicine principles, as most of the studied articles were clinical (92.5%). The results regarding research methods indicated that clinical studies increased their relative weight in the period studied, fundamentally to the detriment of translational studies; research conducted in animals decreased the most (20.7% in 2016 and 7.7% in 2020). The reduction in the number of studies with animals may be owing to the general tendency in the research field to only perform animal experimentation in ethically justified cases where alternatives do not exist (de Boo & Hendriksen, 2005; Gruber & Hartung, 2004). Randomized controlled trials (RCTs) are considered the gold standard in medical research (Concato et al., 2000), and thus, it can be anticipated that a journal such as CIDRR may prioritize this type of research over other types of studies; however, observational studies were predominant during all the series (Fig. 5). The difficulty of elaborating RCTs, which require following strict guidelines (Elliot, 2007), means the researcher must invest significant time, effort, and infrastructure (Institute of Medicine US, 2010). As consequence, few of them have been conducted by a surgical specialist or published in surgical journals (Lee, 2014).

The next level in the pyramid of evidence, observational longitudinal studies, such as case–control or cohort have proven to be as effective in certain situations, as in determining clinical guidelines (Concato et al., 2000). However, in this research, the most notable increase was observed in cross-sectional observational studies (18% in 2016 and 25.6% in 2020), which involve looking at data from a population at one specific point in time. Cross-sectional studies are usually fast and inexpensive to conduct (Wang et al., 2020) and nullify the possibility of patients dropping out from the study, as opposed to longitudinal. These reasons may explain their prevalence in the period studied.

It is possible that the impact of the COVID-19 pandemic, with the increased difficulty of following patients longitudinally due to lockdown, could have influenced these trend variations, as recent literature has noted how pandemic restrictions produced changes in global research (Xu, 2021).

In the present study, some correlations between citations and study parameters were found, which indicates that some variables may influence citation count, as observed by authors in other medical fields (Antoniou et al., 2015; Ruano-Ravina et al., 2016). This study showed a positive correlation between the impact factor and the USA-Canada region, in agreement with the results of Aura-Tormos et al.'s (2019) study on orthodontic journals.

Considering the lines of research, this study observed that the complications topic showed a positive correlation, a matter noticed by some authors (Gupta et al., 2015; Liaw et al., 2015) and can be related to the increasing number of dental implants placed all over the world population. Another positive correlation was found for the sinus elevation topic, a bone regenerative technique for implant placement in the atrophic posterior superior maxilla. This fact may also refer to the increasing amount of implants being placed and it has been noted by citing literature (Bathla et al., 2018).

A comparison of the results obtained in the present study with longer time evolution and with other scientific journals would be convenient to confirm the observed trends.

## Conclusions

This study revealed an increasing trend in Asian research production, particularly Chinese, whereas the research production of European origin showed a decrease. Several publications were produced in the emergent BRICS countries. The most studied topic was "implant/abutment design/treatment of surface" followed by "bone regeneration/expansion" procedures and "prosthetic". Clinical studies, specifically observational cross-sectional studies, increased their relative weight to the detriment of translational ones, and a growing tendency in the relative weight of female authorship was appreciated. Positive correlations were found between impact factors/citations and articles from USA-Canada and EU-Western Europe, and for topics, such as sinus elevation, analysis of performed treatments, and complications, among others.

Recommendations for future research include comparing these results with other implantology-related journals and longer time evolution.

## Appendix

**Table:**

Basic: systematic study directed toward fuller knowledge of the fundamental aspects of phenomena without specific applications toward processes or products in mind (National Science Foundation definition)	Materials: study to test properties of wires or adhesives, not tested in a living organism, including in-vitro studies	
	Cell: bench study involving cell samples	
Translational: research that helps to make findings from basic *science* useful for practical applications (Center for Clinical and Translational Sciences)	Human: involves human extracted teeth	
	Animal: involves animal subjects or animal extracted teeth	
	Theoretical: includes studies on computer modeling, modalities, and laypeople assessing and comparing imaging opinions on esthetics from experts and laypeoples	
Clinical: research involving human volunteers that is intended to add to medical knowledge	Controlled trial: participants receive specific interventions according to the research plan or protocol established by the investigators	Randomized controlled trial: study subjects are randomly allocated to the alternative treatments under study
		Nonrandomized controlled trial: study subjects are not randomly allocated to the alternative treatments under study
	Observational; investigators assess the effects of an intervention on subjects, where assignment of the intervention is outside the investigator's control	Cohort: subjects in treatment groups are followed over time to assess health outcomes
		Case–control; compares subjects with or without a given outcome and determines exposure that led to outcome
		Cast series: reports on treatment course or outcomes for a group of subjects given the same exposure
		Cross-sectional: observation of a population or a representative subset at 1 point in time. including Surveys
